# *CD47* polymorphism for predicting nivolumab benefit in patients with advanced non‑small‑cell lung cancer

**DOI:** 10.3892/ol.2023.13950

**Published:** 2023-07-10

**Authors:** Tatsuya Ogimoto, Hiroaki Ozasa, Hironori Yoshida, Takashi Nomizo, Tomoko Funazo, Hiroshi Yoshida, Kentaro Hashimoto, Kazutaka Hosoya, Masatoshi Yamazoe, Hitomi Ajimizu, Takahiro Tsuji, Yuichi Sakamori, Kiyomitsu Kuninaga, Satoshi Morita, Toyohiro Hirai

**Affiliations:** 1 Department of Respiratory Medicine, Graduate School of Medicine, Kyoto University, Kyoto 606-8507, Japan; 2 Department of Anatomy and Molecular Cell Biology, Graduate School of Medicine, Nagoya University, Nagoya, Aichi 466-8550, Japan; 3 Department of Biomedical Statistics and Bioinformatics, Graduate School of Medicine, Kyoto University, Kyoto 606-8507, Japan

**Keywords:** CD47, single nucleotide polymorphism, non-small-cell lung cancer, immune checkpoint inhibitors, nivolumab

## Abstract

Immune checkpoint inhibitors (ICIs), such as nivolumab, play an essential role in non-small-cell lung cancer (NSCLC) treatment. Programmed death ligand-1 has been used as a predictive biomarker for the efficacy of ICI treatment in patients with NSCLC; however, its predictive value is considered insufficient. Therefore, there is an urgent need for better predictive biomarkers. The present study focused on the CD47 molecule, which is associated with macrophages and tumor immunity. The study aimed to investigate the association between *CD47* single nucleotide polymorphism (SNP) and the therapeutic effect of nivolumab in patients with NSCLC. The *CD47* SNP genotypes and clinical outcomes were retrospectively analyzed in 164 patients with NSCLC treated with nivolumab at Kyoto University Hospital (Kyoto, Japan). Patients with the G/G genotype of the *CD47* SNP rs3804639 had significantly longer progression-free survival than those with the G/T or T/T genotypes [2.6 months vs. 2.1 months, hazard ratio (HR), 0.70; P=0.026]. Moreover, the G/G genotype of the *CD47* SNP rs3804639 was associated with a significantly longer median overall survival than the G/T or T/T genotypes of the *CD47* SNP rs3804639 (24.8 months vs. 12.0 months, HR, 0.64; P=0.021). In conclusion, *CD47* polymorphism may be a novel predictive biomarker of nivolumab efficacy in patients with advanced NSCLC.

## Introduction

Lung cancer is the leading cause of cancer-related deaths worldwide (1). Nivolumab is an immune checkpoint inhibitor (ICI) that has shown significant prognostic benefit in the treatment of non-small-cell lung cancer (NSCLC) (2,3). Based on this benefit, other ICIs, such as pembrolizumab and atezolizumab, have also been used in the treatment of NSCLC, and they have shown prognostic benefits (4,5). Nivolumab combined with ipilimumab has been recently shown to improve NSCLC prognosis (6,7). Although programmed death ligand-1 (PD-L1) expression in tumor cells is mainly used as a biomarker for ICI treatment in patients with NSCLC (8), its predictive ability is inadequate. Tumor mutational burden and microsatellite instability have also been used as biomarkers (9,10), but they have limited predictive ability. Therefore, highly accurate biomarkers are needed to predict the therapeutic effect of ICI treatment in patients with NSCLC.

We have previously reported that *PD-L1* SNPs might predict the therapeutic effect of nivolumab in patients with NSCLC (11,12). Particularly, the *PD-L1* SNP rs822339 predicted prolonged overall survival (OS) in patients with NSCLC treated with nivolumab (13). However, *PD-L1* SNPs alone are still inadequate as predictive biomarkers of nivolumab efficacy because of the lack of improvement in the progressive disease rate in the first 3 months after treatment initiation. PD-1 inhibitors such as nivolumab mainly target cytotoxic T cells. However, some malignant tumors are characterized by cold tumors that lack cytotoxic T cell infiltration (14). In cold tumors, macrophages play an important role in tumor growth and metastasis (15). Macrophages have antigen-presenting capacity and are involved in the immune response (16). Nonetheless, tumor-associated macrophages have reduced antigen-presenting capacity (17).

CD47 was first reported in 1987 as a cell surface antigen encoded by human chromosome 3 (18). It is expressed on red blood cells (RBCs) and plays a role in RBC prevention of phagocytosis by binding to signal regulatory protein α (SIRPα) expressed on macrophages (19). Hence, CD47 is called the ‘do not eat me’ signal (20). CD47 is also expressed on cancer cells and plays a role in preventing phagocytosis by binding to SIRPα expressed in macrophages (21,22). It has been reported that CD47 expression is associated with the prognosis of patients with advanced NSCLC (23). Therefore, CD47 could be a potential target for cancer immunotherapy. For example, it has been reported that blocking CD47 in lung cancer activates macrophage-mediated phagocytosis and enhances the anti-tumor effect (24,25).

Notably, *CD47* SNP rs3804639 has been reported to be associated with the frequency of distant metastases in colorectal cancer (26). The study evaluated *CD47* SNP rs3804639 in 613 patients with colorectal cancer, and patients with the G/G genotype of the *CD47* SNP rs3804639 had a lower frequency of distant metastases than those with the G/T or T/T genotypes of the *CD47* SNP rs3804639. This suggests that the CD47 function of macrophages is lower in the G/G genotype of *CD47* SNP rs3804639. Thus, *CD47* SNP rs3804639 may be a candidate predictive biomarker of nivolumab efficacy in NSCLC.

In this study, we aimed to investigate the association between *CD47* SNP and the therapeutic effect of nivolumab in patients with NSCLC. The study mainly focused on the CD47 molecule associated with macrophages and tumor immunity. We hypothesized that SNPs related to macrophages could also be a predictive biomarker of the therapeutic effect of nivolumab in patients with NSCLC. Further, we hypothesized that the *CD47* SNPs reported in other cancers might be associated with lung cancer. The *CD47* SNP genotypes were measured in patients with NSCLC treated with nivolumab, and the clinical outcomes were analyzed retrospectively.

## Patients and methods

### Study design and patients

**Figure 1.**
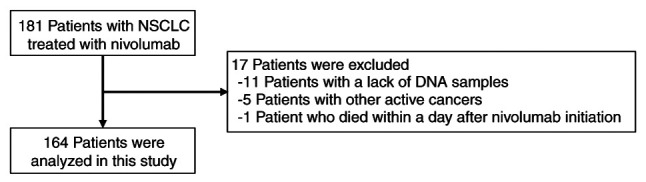
Patient inclusion flow chart. NSCLC, non-small-cell lung cancer.

This retrospective study initially evaluated 181 consecutive patients who were pathologically diagnosed with NSCLC and treated with nivolumab monotherapy at Kyoto University Hospital (Kyoto, Japan) between January 2016 and July 2020. Among them, 17 patients who lacked DNA samples, those with active cancers other than NSCLC, and those who died within 1 day after nivolumab initiation were excluded. Finally, 164 patients were included in the analysis. All patients were treated with nivolumab monotherapy as second- or later-line treatment. Nivolumab was administered at 3 mg/kg or 240 mg every 2 weeks. The patient selection flow chart is shown in Fig. 1.

The association between *CD47* SNP genotypes and progression-free survival (PFS) or OS was analyzed. *PD-L1* SNP was measured in the same patient cohort and combined with the *CD47* SNP for OS analysis. To determine whether the predictive effect of the *CD47* SNP is specific to ICI treatment, 722 patients with advanced NSCLC who received a diagnosis between January 2006 and December 2015 were evaluated. Among them, 478, 139, and 7 patients who lacked DNA samples, received no chemotherapy for NSCLC during data collection, and received ICI during data collection, respectively, were excluded. Finally, 98 patients with available DNA samples and those treated without ICIs were included in the non-ICI cohort for the analysis of the association between *CD47* SNP and clinical outcomes. Data including age, sex, driver mutation status, and survival outcomes were collected from the medical records.

This study, which included both ICI and non-ICI cohorts, was approved by the Ethics Review Board of Kyoto University Hospital (certification number: G0788) and conducted in accordance with the principles of the Declaration of Helsinki. Written informed consent was obtained from all study participants in the ICI and non-ICI cohorts.

### Genotyping and SNP selection

SNP genotyping was performed as described in our previous study in detail (12). Briefly, genomic DNA was extracted from peripheral blood samples using Gene Prep Star NA-480 (Kurabo, Osaka, Japan). Genotyping was performed using the TaqMan genotyping assay (Applied Biosystems, Foster City, CA, USA) and TaqMan genotyping master mix (Applied Biosystems) and analyzed using an Applied Biosystems 7300 Real-Time polymerase chain reaction System (Applied Biosystems). The polymerase chain reaction (PCR) solution consisted of 1 µl of DNA sample, 12.5 µl of TaqMan genotyping master mix, 0.3125 µl of TaqMan genotyping assay primer probe mix, and 11.2 µl of nuclease-free water. The baseline fluorescence measurements were taken at 25°C, followed by the following PCR protocol: incubation of samples at 95°C for 10 min, 40 cycles of denaturation at 92°C for 15 sec, and annealing and extension at 60°C for 1 min, with a final fluorescence measurement at 60°C. SNPs reported to be associated with cancer were selected. Among them, SNPs with minor allele frequencies greater than 0.2 were selected. The TaqMan genotyping assay primer probe mix for the *CD47* SNP rs3804639 (catalog number: 4351379) was purchased from Applied Biosystems.

### Evaluation of nivolumab efficacy and clinical outcomes

Data on clinical characteristics and treatment courses were extracted from the medical records. Radiographic imaging was performed every 6 to 8 weeks. Treatment responses were evaluated according to the Response Evaluation Criteria in Solid Tumors (RECIST) version 1.1 (27). PFS was measured from the initiation of nivolumab administration until the date of disease progression or death. OS was measured from the initiation of nivolumab administration until death or the last follow-up date. Patients with no recorded disease progression at the last follow-up were censored. The cut-off for data collection was August 2021. In the non-ICI cohort, OS was measured from the initiation of cytotoxic chemotherapy or tyrosine kinase inhibitors until death or the last follow-up date. The cut-off for data collection in the non-ICI cohort was December 2016.

### Statistical analysis

Patient characteristics were compared by genotype using the following statistical tests: Kruskal-Wallis test was used for age, and the Fisher's exact test was used for all other variables. PFS and OS survival curves were generated using the Kaplan-Meier method and compared by genotype using the log-rank test. Univariate and multivariate analyses were performed using the Cox regression model to estimate hazard ratios (HRs) with 95% confidence intervals (CIs). P<0.05 was considered to indicate a statistically significant difference. All statistical analyses were performed using the JMP Pro statistical software version 15.2.0 (SAS Institute, Cary, NC, USA).

## Results

### Patient characteristics and clinical outcomes

The ICI cohort had a median age of 69 years (range, 30–85 years), and 105 patients (64.0%) were male. The patient characteristics according to the *CD47* SNP rs3804639 genotype are summarized in Table I. A total of 150 patients (91.5%) had an Eastern Cooperative Oncology Group (ECOG) performance status (PS) of 0 or 1, and 116 (70.7%) patients had adenocarcinoma. Further, 35 (21.3%) patients harbored epidermal growth factor receptor (EGFR) mutations, and 34 (20.7%) patients had liver metastases. Eighty two (50.0%) patients were administered nivolumab as second-line treatment, while the remaining patients received nivolumab as third- or later-line treatment. *CD47* SNP rs3804639 genotypes were not associated with clinical factors. With respect to clinical outcomes, the overall response rate was 14.6%, and the disease control rate was 48.2% in all patient populations. The median PFS was 2.2 (95% CI, 1.9–2.8) months, and the median OS was 16.1 (95% CI, 11.1–23.0) months in the patient populations.

### Association between clinical outcomes and CD47 SNP rs3804639

**Figure 2.**
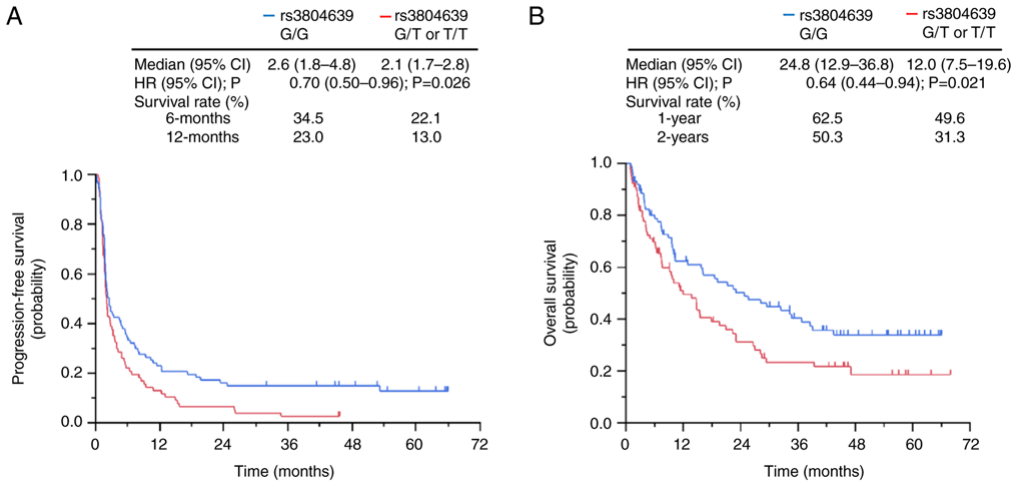
Kaplan-Meier curves after nivolumab administration according to CD47 rs3804639 genotypes. (A) Progression-free survival and (B) overall survival. CI, confidence interval; HR, hazard ratio.

Patients with the G/G genotype of the *CD47* SNP rs3804639 had significantly longer PFS than those with the G/T or T/T genotypes of the *CD47* SNP rs3804639 (2.6 months vs. 2.1 months, HR, 0.70; P=0.026; Fig. 2A). In addition, patients with the G/G genotype of *CD47* SNP rs3804639 had a significantly longer OS than had those with the G/T or T/T genotypes of *CD47* SNP rs3804639 (24.8 months vs. 12.0 months, HR, 0.64; P=0.021; Fig. 2B). The 1- and 2-year survival rate of patients with the G/G genotype of *CD47* SNP rs3804639 were higher than those of patients with the G/T or T/T genotypes of *CD47* SNP rs3804639 (1-year survival rate, 62.5% vs. 49.6%; 2-year survival rate, 50.3% vs. 31.3%; Fig. 2B).

### Influencing factors of OS

Univariate analysis showed that *CD47* SNP rs3804639, ECOG PS, and the presence or absence of liver metastases were associated with OS. Multivariate analysis showed that *CD47* SNP rs3804639 was independently associated with OS. The results of the uni- and multivariate analyses are shown in Table II. The finding that PS and liver metastases are associated with the prognosis of patients with NSCLC treated with nivolumab is consistent to that of previous reports (28,29).

### Association between clinical outcomes and combination of CD47 SNP rs3804639 and PD-L1 SNP rs822339

**Figure 3.**
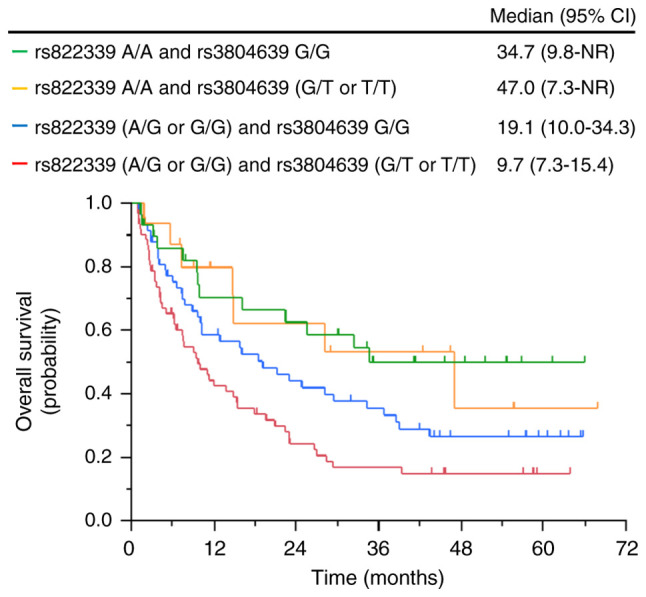
Kaplan-Meier curves of overall survival according to the combination of PD-L1 rs822339 genotypes and CD47 rs3804639 genotypes. CI, confidence interval; NR, not reached.

The *PD-L1* SNP rs822339 has been previously shown to predict survival outcomes in patients with NSCLC treated with nivolumab (13). In this study, even in patients with the A/G or G/G genotypes of the *PD-L1* SNP rs822339, which were reported to be associated with poor prognosis, patients with the G/G genotype of the *CD47* SNP rs3804639 had longer OS than those with the G/T or T/T genotypes of the *CD47* SNP rs3804639 (19.1 months vs. 9.7 months) (Fig. 3).

### Association between clinical outcomes and CD47 SNP rs3804639 in the non-ICI cohort

**Figure 4.**
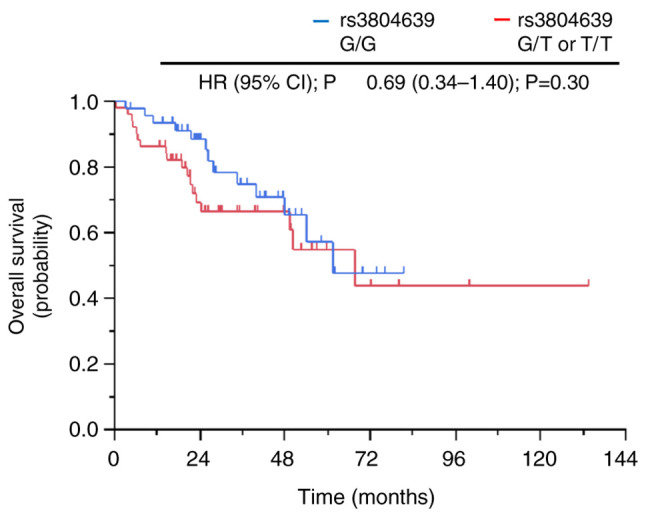
Kaplan-Meier curves of overall survival according to CD47 rs3804639 genotypes in the non-immune checkpoint inhibitor cohort. HR, hazard ratio; CI, confidence interval.

The baseline characteristics according to the *CD47* SNP rs3804639 genotype in the non-ICI cohort are summarized in Table SI. The median patient age was 66 years (range, 32–89 years), and 55 patients (56.1%) were male. There were 92 (93.9%) patients with an ECOG PS of 0 or 1, and 89 (90.8%) patients had adenocarcinoma. Further, 61 (62.2%) patients harbored EGFR mutations, and 3 (3.1%) patients had liver metastases. The genotypes of *CD47* SNP rs3804639 were not associated with clinical factors. None of the patients in this cohort were administered nivolumab or other ICIs during data collection. Regarding *CD47* SNP rs3804639, there was no significant difference in OS between patients with the G/G genotype and those with the G/T or T/T genotypes (61.5 months vs. 67.7 months, HR, 0.69; P=0.30; Fig. 4).

## Discussion

Accurate predictive biomarkers of ICI efficacy in NSCLC are lacking. This study found that the G/G genotype of the *CD47* SNP rs3804639 was associated with significantly longer survival in patients with advanced NSCLC treated with nivolumab. In addition, patients with the A/G or G/G genotypes of *PD-L1* SNP rs822339 had longer survival than those with the G/G genotype of *CD47* SNP rs3804639. Thus, *CD47* SNP rs3804639 might be a predictive biomarker of nivolumab efficacy in patients with advanced NSCLC. To our best knowledge, this study is the first to show that *CD47* SNP is associated with survival outcomes in patients with advanced NSCLC treated with nivolumab.

The current study also found that the G/G genotype of *CD47* SNP rs3804639 has a survival advantage. However, this survival advantage was not observed in the non-ICI cohort, which involved patients who did not receive nivolumab or other ICIs (Fig. 4). This indicates that *CD47* SNP rs3804639 can be a predictive biomarker of nivolumab efficacy but not of prognosis in patients with advanced NSCLC. This result is similar to that of a previous report showing that the G/G genotype of the *CD47* SNP rs3804639 is associated with a lower frequency of distant metastases in colorectal cancer (26); therefore, the G/G genotype of *CD47* SNP rs3804639 is suggested to be functional in that it reduces cancer progression by suppressing CD47 function.

The *CD47* SNP rs3804639 located in the intron region of the gene has a relatively small effect on the protein function compared to that of SNPs in the exon region. However, SNPs in the intron region can still regulate the protein function by regulating alternative splicing (30). CD47 is expressed on various immune cells other than macrophages; for example, CD47 expressed on T cells has been associated with enhanced T cell immune responses (31). To evaluate the role of *CD47* SNP rs3804639, we used quantitative trait locus (QTL) data from the Genotype-Tissue Expression Program. Data on the expression QTL, which are the quantitative effects of SNPs on gene expression, were unavailable for *CD47* SNP rs3804639. However, data on the splicing QTL (sQTL), which are the quantitative effects of SNPs on alternative splicing, were available for *CD47* SNP rs3804639. The sQTL for the *CD47* SNP rs3804639 in the esophagus and skin showed different trends depending on the genotype. The sQTL data from these organs suggest the possibility of a relationship between *CD47* SNP rs3804639 and the regulation of alternative splicing. In the future, when surgical specimens of lung cancer are obtained, we hope to investigate the association between *CD47* SNP rs3804639 and CD47 protein expression by staining for the CD47 protein in tumors and surrounding immune cells.

Tumor immunity is caused not only by cytotoxic T lymphocytes associated with PD-1/PD-L1 pathways but also by other immune-related cells (e.g., macrophages and dendritic cells) (32.33). This could be the reason for the insufficient predictive ability of *PD-L1* SNPs alone as biomarkers of nivolumab efficacy in patients with NSCLC. In this study, we focused on CD47 based on previous evidence that high CD47 expression is associated with resistance to nivolumab treatment (34). The results indicated an association between the *CD47* SNP and therapeutic effect of nivolumab, representing the same association between CD47 expression and nivolumab resistance in the above study. Tumor-associated macrophages have also been reported to express PD-1 (35), which might influence differences in macrophage function and nivolumab efficacy.

This study also found that the combination of *CD47* and *PD-L1* SNP has better predictive accuracy for nivolumab efficacy. Particularly, patients with the G/G genotype of *CD47* SNP rs3804639 treated with nivolumab had better prognoses, even patients with the A/G or G/G genotypes of *PD-L1* SNP rs822339, which have been previously reported to be associated with poor prognoses (13). This result suggests that in these patients, CD47 function is suppressed and antigen-presenting capacity is increased, but PD-L1 function is impaired. This result might also indicate that nivolumab would be effective for these patients, even in those with cold tumors. Although these patients have impaired PD-L1 function, nivolumab could be effective if CD47 function is suppressed and the antigen-presenting capacity of macrophages is restored. Thus, *CD47* SNP rs3804639 could be a predictive biomarker of nivolumab treatment in PD-L1-independent tumors.

Given these results, other immune-related SNPs could predict the effect of nivolumab treatment with higher accuracy when combined with the *PD-L1* SNP or *CD47* SNP. We will further investigate SNPs that could predict the effect of nivolumab treatment, using more high-throughput methods (e.g., a genome-wide association study). Clinical trials targeting CD47 as a potential therapeutic target are in progress. For instance, phase I trials on Hu5F9-G4 (5F9), a humanized IgG4 antibody targeting CD47, for lymphoma, lung cancer, and other cancers have shown its efficacy (36,37). A phase II study of anti-CD47 antibodies in solid tumors is also in progress. When CD47-targeted therapies became available in clinical practice, we will investigate the capability of *CD47* SNP rs3804639 for predicting treatment response to CD47-targeted therapies.

Our study has some limitations. First, this was a single-center retrospective cohort study with a small sample size. Large multicenter cohorts would provide more reliable results. We are currently planning studies to evaluate the relevance of the effect of nivolumab treatment and SNPs in a multicenter cohort (UMIN000033839). Second, a *CD47* SNP that has been reported to be associated with other cancers was selected, and other SNPs were not investigated. We plan to investigate the relationship between various SNPs and the therapeutic effect of nivolumab treatment in a multicenter cohort.

In conclusion, *CD47* polymorphism is associated with survival outcomes in patients with advanced NSCLC treated with nivolumab. The *CD47* SNP alone or in combination with the *PD-L1* SNP might be helpful predictive biomarkers of nivolumab treatment in patients with advanced NSCLC.

## Supplementary Material

Supporting Data

## Figures and Tables

**Table I.** Patient characteristics according to *CD47* rs3804639 genotype

		*CD47* rs3804639			
			
Characteristic	Overall (n=164)	G/G (n=87)	G/T (n=70)	T/T (n=7)	P-value^a^
Median age, years (range)	69 (30–85)	69 (30–83)	70 (33–85)	70 (64–79)	0.23
Sex, n (%)					0.75
Female	59 (36.0%)	30 (34.5%)	27 (38.6%)	2 (28.6%)	
Male	105 (64.0%)	57 (65.5%)	43 (61.4%)	5 (71.4%)	
Smoking status, n (%)					0.32
Current/former	113 (68.9%)	63 (72.4%)	45 (64.3%)	5 (71.4%)	
Never	51 (31.1%)	24 (27.6%)	25 (35.7%)	2 (28.6%)	
ECOG PS, n (%)					
0-1	150 (91.5%)	82 (94.3%)	61 (87.1%)	7 (100%)	0.26
2-3	14 (8.5%)	5 (5.7%)	9 (12.9%)	0 (0%)	
Histology, n (%)					0.37
Adenocarcinoma	116 (70.7%)	57 (65.5%)	54 (77.1%)	5 (71.4%)	
Squamous	35 (21.3%)	20 (23.0%)	13 (18.6%)	2 (28.6%)	
Others	13 (7.9%)	10 (11.5%)	3 (4.3%)	0 (0%)	
EGFR mutation, n (%)					0.35
Positive	35 (21.3%)	16 (18.4%)	19 (27.1%)	0 (0%)	
Negative or unknown	129 (78.7%)	71 (81.6%)	51 (72.9%)	7 (100%)	
PD-L1 status, n (%)					0.06
Positive	42 (25.6%)	27 (31.0%)	14 (20.0%)	1 (14.3%)	
Negative	45 (27.4%)	21 (24.1%)	19 (27.1%)	5 (71.4%)	
Unknown	77 (47.0%)	39 (44.8%)	37 (52.9%)	1 (14.3%)	
Treatment line, n (%)					1.00
Second	82 (50.0%)	44 (50.6%)	32 (45.7%)	6 (85.7%)	
Third or later	82 (50.0%)	43 (49.4%)	38 (54.3%)	1 (14.3%)	
Liver metastasis, n (%)					1.00
Positive	34 (20.7%)	18 (20.7%)	13 (18.6%)	3 (42.9%)	
Negative	130 (79.3%)	69 (79.3%)	57 (81.4%)	4 (57.1%)	

a P-value for comparison between patients with the G/G genotype and those with the G/T or G/G genotype of rs3804639. P-value for age is across all three groups. ECOG, Eastern Cooperative Oncology Group; PS, performance status; EGFR, epidermal growth factor receptor.

**Table II.** Univariate and multivariate analyses of influencing factors of overall survival.

	Univariate			Multivariate		
		
Variable	HR	95% CI	P-value	HR	95% CI	P-value
Age (≥75 vs. <75 years)	1.03	0.66–1.63	0.89	1.15	0.69–1.91	0.6
Smoking status (current/former vs. never)	0.86	0.57–1.29	0.47	0.79	0.47–1.34	0.39
ECOG PS (≥2 vs. 0–1)	3.43	1.90–6.18	<0.001	3.49	1.86–6.53	<0.001
Histology (non-Sq vs. Sq)	0.96	0.61–1.49	0.84	0.8	0.49–1.32	0.38
EGFR mutation (positive vs. negative or unknown)	1.01	0.63–1.63	0.96	0.85	0.46–1.56	0.59
Treatment line (2nd vs. ≥3rd)	0.78	0.53–1.14	0.19	0.76	0.48–1.20	0.24
Liver metastasis (positive vs. negative)	1.82	1.17–2.84	0.008	2.19	1.37–3.51	0.001
*CD47* rs3804639 (G/G vs. G/T or T/T)	0.64	0.44–0.94	0.022	0.66	0.44–0.98	0.04

ECOG, Eastern Cooperative Oncology Group; PS, performance status; Sq, squamous cell carcinoma; EGFR, epidermal growth factor receptor; HR, hazard ratio; CI, confidence interval.

## Data Availability

The datasets used and/or analyzed during the current study are available from the corresponding author on reasonable request.
