# Supplementary material for: CD47 polymorphism for predicting nivolumab benefit in patients with advanced non‑small‑cell lung cancer
Source: Oncol Lett. 2023 Jul 10;26(2):364. doi: 10.3892/ol.2023.13950 (PMC10398628; doi:10.3892/ol.2023.13950)
Supplement: Supporting Data [file Supplementary_Data.pdf]

Table SI. Patient characteristics according to *CD47* rs3804639 genotype in the non-ICI cohort.

Characteristic	Overall (n=98)	*CD47* rs3804639 G/G (n=47)	G/T (n=45)	T/T (n=6)	P-value[a]
Median age, years(range)	66 (32-89)	67 (36-89)	66 (35-86)	60 (32-73)	0.32
Sex, n (%)					0.22
Female	43 (43.9%)	24 (51.1%)	16 (35.6%)	3 (50.0%)	
Male	55 (56.1%)	23 (48.9%)	29 (64.4%)	3 (50.0%)	
Smoking status, n (%)					1.00
Current/former	48 (49.0%)	23 (48.9%)	23 (51.1%)	2 (33.3%)	
Never	50 (51.0%)	24 (51.1%)	22 (48.9%)	4 (66.7%)	
ECOG PS, n (%)					0.21
0-1	92 (93.9%)	46 (97.9%)	41 (91.1%)	5 (83.3%)	
2-4	6 (6.1%)	1 (2.1%)	4 (8.9%)	1 (16.7%)	
Histology, n (%)					0.25
Adenocarcinoma	89 (90.8%)	45 (95.7%)	38 (84.4%)	6 (100%)	
Squamous	4 (4.1%)	0 (0%)	4 (8.9%)	0 (0%)	
Others	5 (5.1%)	2 (4.3%)	3 (6.7%)	0 (0%)	
EGFR mutation, n (%)					0.21
Positive	61 (62.2%)	26 (55.3%)	31 (68.9%)	4 (66.7%)	

Negative or unknown	37 (37.8%)	21 (44.7%)	14 (31.1%)	2 (33.3%)	
First-line treatment, n (%)					0.69
Cytotoxic chemotherapy	47 (48.0%)	24 (51.1%)	20 (44.4%)	3 (50.0%)	
EGFR or other TKI	51 (52.0%)	23 (48.9%)	25 (55.6%)	3 (50.0%)	
Liver metastasis, n (%)					0.61
Positive	3 (3.1%)	2 (4.3%)	1 (2.2%)	0 (0%)	
Negative	95 (96.9%)	45 (95.7%)	44 (97.8%)	6 (100%)	

[a]P-value for comparison between patients with the G/G genotype and those with the G/T or G/G genotype of rs3804639.P-value for age is across all three groups.ECOG, Eastern Cooperative Oncology Group; PS, performance status; EGFR, epidermal growth factor receptor; TKI, tyrosine kinase inhibitor.
